# Patient and health system delay among patients with pulmonary tuberculosis in Beira city, Mozambique

**DOI:** 10.1186/1471-2458-13-559

**Published:** 2013-06-07

**Authors:** Abuchahama Saifodine, Paula Samo Gudo, Mohsin Sidat, James Black

**Affiliations:** 1 Community Health Department, Faculty of Medicine, Eduardo Mondlane University, Maputo, Mozambique; 2 National TB Control Programme, Maputo, Mozambique; 3 Nossal Institute for Global Health, Melbourne School of Population and Global Health, University of Melbourne, Melbourne, Australia

## Abstract

**Background:**

TB control is based on the rapid identification of cases and their effective treatment. However, many studies have shown that there are important delays in diagnosis and treatment of patients with TB. The purpose of this study was to assess the prevalence of and identify risk factors associated with patient delay and health system delay among newly diagnosed patients with pulmonary TB.

**Methods:**

A cross sectional study was carried out in Beira city, Mozambique between September 2009 and February 2010. Patients in the first month of treatment were consecutively selected to this study if they had a diagnosis of pulmonary TB, had no history of previous TB treatment, and were 18 years or older and provided informed consent. Data was obtained through a questionnaire administered to the patients and from patients’ files.

**Results:**

Among the 622 patients included in the study the median age was 32 years (interquartile range, 26–40) and 272 (43.7%) were females. The median total delay, patient delay and health system delay was 150 days (interquartile range, 91–240), 61 days (28–113) and 62 days (37–120), respectively. The contribution of patient delay and health system delay to total delay was similar. Farming, visiting first a traditional healer, low TB knowledge and coexistence of a chronic disease were associated with increased patient delay. More than two visits to a health facility, farming and coexistence of a chronic disease were associated with increased health system delay.

**Conclusions:**

This study revealed a long total delay with a similar contribution of patient delay and health system delay. To reduce the total delay in this setting we need a combination of interventions to encourage patients to seek appropriate health care earlier and to expedite TB diagnosis within the health care system.

## Background

Tuberculosis (TB) continues to represent an important public health problem worldwide and Mozambique is one of the most affected countries [1]. TB control is based on the rapid identification of cases and their effective treatment. The rapid identification of cases depends, on one side, on patients promptly recognizing TB symptoms and seeking appropriate health care, and on the other side, on the capacity of the health care system to diagnose the disease.

The results of several studies have shown that there are important delays in TB diagnosis and treatment and those delays are caused both by patients and the health care system [2-22]. Delays in diagnosis and treatment of TB have important individual and public health implications. At individual level, patients experiencing long delays in initiation of treatment may have an increased risk of developing severe disease and death [23,24]. At a public health level, these delays may increase TB incidence by increasing the risk of transmission [4,25]. The purpose of this study was to assess the prevalence of and identify risk factors associated with patient delay and health system delay among newly diagnosed patients with pulmonary TB. The study also compares the results by sputum smear status.

## Methods

### Ethical considerations

This study was approved by the Human Research Ethics Committee of The University of Melbourne, by the Mozambican National Bioethics committee and by the Mozambican Ministry of Health.

### Setting

This study was carried out in Beira city, Mozambique during the period from September 2009 to February 2010. The city has a population of 431,965 inhabitants with 16 health facilities including one tertiary hospital and three private clinics. TB drugs are only available at six government health centres (referred to as TB clinics) and at the tertiary hospital. This study was based in the five TB clinics that accounted for more than 90% of the TB notifications in Beira city. TB diagnosis was based on sputum smear microscopy and chest X-rays. Chest X-rays were only performed at the tertiary hospital and TB culture was not available at the time of this study.

### Patients

Patients in the first month of treatment were consecutively selected to this study if they had a diagnosis of pulmonary TB, had no history of previous TB treatment, and were 18 years or older and provided informed consent. Socio-demographic and clinical data was collected through a questionnaire, which was administered in Portuguese through a face-to-face interview. Results of routine HIV and sputum smear microscopy tests were retrieved upon authorization from the patients. An additional file provides an English translation of the questions included in the questionnaire [see Additional file 1].

### Definitions of delay

Patient delay was defined as the time interval between debut of symptoms and first contact with the health care system; health system delay was defined as the time interval between first contact with the health care system and initiation of TB treatment. Delay was measured in days. To help patients recall dates of health seeking encounters and duration of symptoms we used a locally adapted calendar and reviewed medical prescriptions and other medical documents. The calendar contained political events, personal and family events, holidays, and religious celebration days.

### Statistical analysis

Descriptive statistics were used to summarize the data. To assess the relationship between two variables, the chi-square test of association or the rank sum test were used, as indicated. The level of TB knowledge and perceived TB stigma was assessed by constructing a summation score that has been described elsewhere [26]. TB knowledge was assessed by asking patients the following questions: is TB a contagious disease? Is TB a curable disease? What is the duration of TB treatment? Can you name at least one anti-tuberculosis drug? The answers were recorded as correct, incorrect or don’t know. Questions were weighted equally and each correct answer was assigned one point so a higher score was an indication of good TB knowledge. Incorrect and don’t know answers were assigned zero points. A similar process was used to construct a score for perceived TB stigma, using the following statements: people with TB are ashamed of having the disease; TB affects the relationship with people; people with TB prefer to live isolated; TB affects the relationship between husband and wife; TB affects the relationship with other family members; a woman with TB can cook for the family; a person with TB can share his plate, cup and spoons with other members of the family; a woman with TB cannot have children; a woman with TB can breastfeed. Patients had three options to answer these questions: agree, disagree or don’t know. Questions were equally weighted and each answer indicating a perception related to stigma was assigned one point; otherwise the answer received zero points. To ensure consistency some items were reverse coded. For knowledge a score smaller than three was considered low TB knowledge. For perceived stigma a score above the median was considered high level of perceived TB stigma. Inadequate physical access (from now on referred to as inadequate access) to health services was defined as taking more than one hour to reach a healthcare facility on foot or by bicycle. The analysis of risk factors associated with patient and health system delay was carried out using logistic regression. For this purpose, the outcome variables were dichotomized using the median as the cut-off point. Data analysis was performed using *Stata Intercooled, version 10* (Stata Corporation, College Station, Texas).

## Results

### Characteristics of the patients

The study included 622 patients from which 312 (50.2%) were sputum smear-positive and 310 (49.8%) were sputum smear-negative patients. The median age was 32 years (interquartile range, 26–40) and there were 272 females (43.7%). Males were older than females, median age of 34 years versus 30 years, respectively (rank sum test, p < 0.001).

Table 1 shows the distribution of several patients’ characteristics. HIV results were available for 617 patients. The proportion of HIV infection was higher among smear-negative patients compared to smear-positive patients, 79.8 percent versus 65.2 percent, respectively (chi-square test of association, p < 0.01). The proportion of patients with adequate TB knowledge was higher among HIV-positive patients compared to HIV-negative patients, 69.1 percent versus 30.9 percent, respectively (chi-square test of association, p < 0.05). Among 617 patients 113 (18.3%) had inadequate access to a health facility.

**Table 1:** Demographic and clinical characteristics of patients

Characteristic*	**n (%)**
Male sex	350 (56.3)
Non-farmers	563 (91.7)
High TB knowledge	366 (58.8)
Low perceived TB stigma	242 (38.9)
HIV negative	170 (27.6)†
Never smokers	464 (74.6)
Never drinkers	253 (40.7)
No household TB contact	441 (70.9)
No coexistence of a chronic disease	494 (79.4)

*Only the reference category is shown.

†HIV results were available for 617 patients.

### Duration and distribution of delay

The median total delay, patient delay and health system delay was 150 days (interquartile range, 91–240), 61 days (28–113) and 62 days (37–120), respectively. Total delay among patients with smear-negative disease and smear-positive disease was 180 days and 123 days, respectively (rank sum test, p < 0.01). Patients with smear-negative disease had longer health system delay than patients with smear-positive disease; 70 days versus 53 days respectively (rank sum test, p < 0.001). There were no differences in the duration of patient delay according to sputum smear status (p = 0.168). The median time between TB diagnosis and initiation of TB treatment was one day (interquartile range, 0–3).

### Patient delay

The main reasons for seeking health care were cough (88.3%), fever (56.9%), weight loss (14.3%) and chest pain (13.2%). Among the 618 patients with these data 406 (65.7%) first visited a public health facility, 154 (24.9%) first sought help from a traditional healer, 34 (5.5%) went to a pharmacy, 16 (2.6%) self-medicated, and eight (1.3%) went to a private clinic. Among the 154 patients who reported first visiting a traditional healer, 138 (89.6%) did so because they thought it was a “traditional” rather than a medical problem; 13 (8.4%) said that the health facility was too far and 3 (1.9%) considered the waiting time at the health facility too long. Among 596 patients who indicated their perception in relation to delay in seeking health care, 224 (37.6%) said that patients delay because they think “The symptoms will disappear”, 102 (17.1%) referred to “Fear of the diagnosis”, 86 (14.4%) said “TB is a traditional disease”, 15 (2.5%) referred to poor quality of the health services and 15 (2.5%) referred to lack of money to pay for transportation costs; 154 (25.8%) patients said that patients do not delay.

Table 2 describes the results of univariable analysis for those variables included in the multivariable analysis. Other variables considered in the univariable analysis that were not selected for the multivariable analysis included female sex (odds ratio = 1.2; 95% CI: 0.8-1.7), perceived TB stigma (1.0; 0.7-1.5), current or ever drinking (1.1; 0.8-1.6) and history of household TB contact (1.2; 0.8-1.7). Table 3 describes the results of multivariable analysis for patient delay. As shown in Table 3, farming was associated with increased patient delay in all groups of patients. Visiting first a traditional healer and coexistence of a chronic disease were associated with increased patient delay among smear-positive patients, while low TB knowledge was associated with increased patient delay among smear-negative patients. HIV infection was associated with shorter patient delay among smear-positive patients.

**Table 2:** Results of the univariable analysis of patient delay across different groups of patients, Beira city, 2009

**Risk factor**	**All PTB patients**			**New smear-positive**			**New smear-negative**		
	**n**	**UOR**	**95% CI**	**n**	**UOR**	**95% CI**	**n**	**UOR**	**95% CI**
Age (>32 years)	250	1.5	1.1-2.2	106	1.7	1.0-2.8	144	1.2	0.8-2.0
Female sex	272	1.2	0.8-1.7	129	1.2	0.7-2.0	143	1.1	0.7-1.8
Farming	51	4.2	2.1-8.6	21	4.2	1.5-11.8	30	4.0	1.5-10.8
Primary education or less	356	1.3	0.9-1.9	182	0.9	0.5-1.5	174	2.0	1.2-3.4
Visiting first a traditional healer	148	2.2	1.5-3.3	68	2.4	1.4-4.2	80	2.0	1.1-3.5
Low level of TB knowledge	227	1.8	1.3-2.6	113	1.4	0.8-2.2	114	2.5	1.5-4.1
HIV infection	393	0.8	0.6-1.2	175	0.6	0.4-1.0	218	1.0	0.6-1.9
Current or ever smoking	137	1.3	0.9-1.9	74	1.4	0.8-2.3	63	1.3	0.7-2.3
Presence of a chronic disease	120	1.6	1.0-2.4	54	1.6	0.9-3.0	66	1.4	0.8-2.6
Inadequate access to health services	103	1.6	1.0-2.4	49	1.6	0.8-2.9	54	1.5	0.8-2.9

*PTB* pulmonary TB, *UOR* unadjusted odds ratio, 95% CI: 95% confidence interval.

**Table 3:** Results of the multivariable analysis of patient delay across different groups of patients, Beira city, 2009

**Risk factor**	**All PTB patients**			**New smear-positive**			**New smear-negative**		
	**n**	**AOR**	**95%CI**	**n**	**AOR**	**95%CI**	**n**	**AOR**	**95%CI**
Age (>32 years)	250	1.35	0.94-1.94	106	1.48	0.86-2.55	144	1.04	0.62-1.75
Farming	51	3.65	1.74-7.67	21	4.19	1.38-12.70	30	2.99	1.07-8.38
Primary education or less	356	0.82	0.55-1.23	182	0.57	0.31-1.04	174	1.28	0.73-2.24
Visiting first a traditional healer	148	2.05	1.35-3.11	68	2.36	1.28-4.34	80	1.67	0.92-3.04
Low level of TB knowledge	227	1.64	1.12-2.40	113	1.45	0.82-2.57	114	2.03	1.18-3.51
HIV infection	393	0.70	0.46-1.06	175	0.49	0.27-0.88	218	0.87	0.45-1.68
Current or ever smoker	137	1.44	0.95-2.18	74	1.61	0.89-2.90	63	1.39	0.74-2.58
Coexistence of a chronic disease	120	1.76	1.12-2.77	54	2.34	1.18-4.64	66	1.45	0.77-2.72
Inadequate access to health services	103	1.45	0.91-2.32	49	1.54	0.77-3.08	54	1.32	0.68-2.59
Total number of observations	539			266			273		

*PTB* pulmonary TB, *AOR* adjusted odds ratio, 95% CI 95% confidence interval.

### Health system delay

Of the 622 patients 606 (97.4%) were diagnosed with TB at public health facilities. Among these 606 patients, 434 (71.6%) were diagnosed at primary health clinics and the remaining patients were diagnosed at the tertiary hospital; 439 (72.4%) were diagnosed by a nurse or medical assistant, 161 (26.6%) were diagnosed by a medical doctor and for the remaining six patients this information was not recorded.

Table 4 describes the results of univariable analysis for those variables included in the multivariable analysis. Other variables considered in the univariable analysis that were not selected for the multivariable analysis included health worker who made the TB diagnosis (odds ratio = 1.0; 95% CI: 0.7-1.4), TB knowledge (0.9; 0.7-1.3), perceived TB stigma (0.9; 0.6-1.2), current or ever smoking (0.9; 0.6-1.3), current or ever drinking (1.1; 0.8-1.5) and access to health services (1.0; 0.7-1.5) and place where TB diagnosis was made (1.1; 0.8-1.6). Table 5 describe the results of the multivariable analysis for health system delay. As shown in Table 5, making more than two visits to a health facility was associated with at least a six-fold increase in health system delay. Farming was associated with increased health system delay among new smear-positive patients while coexistence of a chronic disease was associated with increased health system delay among smear-negative patients.

**Table 4:** Results of the univariable analysis of health system delay across different groups of patients, Beira city, 2009

**Risk factor**	**All PTB patients**			**New smear-positive**			**New smear-negative**		
	**n**	**UOR**	**95% CI**	**n**	**UOR**	**95% CI**	**n**	**UOR**	**95% CI**
Age (>32 years)	265	1.0	0.7-1.4	115	0.9	0.5-1.4	150	1.0	0.6-1.6
Female sex	259	1.3	0.9-1.8	126	1.6	1.0-2.6	133	1.0	0.6-1.6
Farming	49	1.8	1.0-3.3	21	2.6	1.0-6.8	28	1.2	0.6-2.7
Primary education or less	380	1.0	0.7-1.4	201	1.4	0.8-2.2	179	0.8	0.5-1.3
More than 2 visits to a health facility	434	16.6	9.4-29.3	207	11.4	5.7-22.7	227	29.1	10.1-83.5
Negative sputum smear result	284	1.6	1.2-2.2	NA	NA	NA	NA	NA	NA
HIV infection	421	1.9	1.3-2.7	195	2.0	1.2-3.3	226	1.4	0.8-2.5
History of household TB contact	181	1.4	1.0-2.0	94	1.4	0.8-2.2	81	1.6	0.9-2.7
Coexistence of a chronic disease	122	3.1	2.0-4.7	56	2.2	1.2-4.0	66	4.2	2.2-8.2

*PTB* pulmonary TB, *UOR* unadjusted odds ratio, 95% CI 95% confidence interval, *NA* not applicable.

**Table 5:** Results of the multivariable analysis of health system delay across different groups of patients, Beira city, 2009

**Risk factor**	**All PTB patients**			**New smear-positive**			**New smear-negative**		
	**n**	**AOR**	**95%CI**	**n**	**AOR**	**95%CI**	**n**	**AOR**	**95%CI**
Age (>32 years)	265	0.88	0.59-1.33	115	0.84	0.48-1.47	150	0.93	0.51-1.71
Female sex	259	0.88	0.58-1.32	126	1.10	0.63-1.93	133	0.74	0.40-1.38
Farming	49	2.19	1.02-4.72	21	4.85	1.47-15.93	28	1.20	0.44-3.28
Primary education or less	380	0.98	0.65-1.48	201	1.27	0.71-2.26	179	0.77	0.42-1.43
More than 2 visits to a health facility	434	16.58	9.21-29.84	207	13.00	6.19-27.29	227	31.03	10.39-92.69
Negative sputum smear result	284	1.24	0.84-1.83	NA	NA	NA	NA	NA	NA
HIV infection	421	1.46	0.94-2.26	195	1.79	1.00-3.19	226	1.22	0.62-2.42
History of household TB contact	175	1.18	0.77-1.81	94	1.20	0.67-2.13	81	1.14	0.60-2.16
Coexistence of a chronic disease	122	2.95	1.74-5.01	56	1.85	0.90-3.80	66	4.73	2.06-10.88
Total number of observations	586			302			284		

*PTB* pulmonary TB, *AOR* adjusted odds ratio 95%, *CI* 95% confidence interval, *NA* not applicable.

## Discussion

This is the first study from Mozambique that assessed the prevalence of and the risk factors associated with patient and health system delays. The median total delay of 150 days was similar to the delay observed in Tanzania (136 days) [27] and in Ghana (120 days) [28]. However, it was much higher than the delay observed in other African countries such as Uganda (84 days) [29], South Africa (60 days) [16], rural Botswana (84 days) [19], and Nigeria (70 days) [30]. This finding is particularly concerning as the majority of the patient delay and health system delay studies carried out elsewhere were conducted in referral hospitals, where longer patient and health system delays are expected. Our study was carried out in primary health clinics. The fact that patient delay and health system delay had a similar contribution to total delay indicates that there are many opportunities to reduce total delay by intervening both at the community and in the health care system.

### Patient delay

The median patient delay of 60 days observed in our study was similar to that observed in Addis Ababa, Ethiopia [31] and Lagos, Nigeria [30]. However, it was higher than the delay observed in Uganda [29], South Africa [15], and Botswana [19].

The association between patient delay and farming observed in this study is consistent with findings from studies carried out elsewhere [11,29]. It has been suggested that the increased delay observed among farmers may be related to their socio-economic condition, specifically low levels of education and high levels of poverty [29]. The delay observed among farmers in our study may be related to the time farmers spend away from the city working in their fields. In addition, farming is a non-salaried and seasonally dependant work and this may force farmers to delay treatment seeking in the busy times even if they are very ill.

The increased delay among patients visiting first a traditional healer is also consistent with findings from other studies [19,26,32-34]. Our results indicate that traditional healers play an important role in the provision of TB care in this population, as a quarter of patients referred to traditional healers as the first contact of care and a significant proportion of patients indicated that TB is a traditional disease. This also indicates that there is a high level of misconceptions about TB in this population.

The increase in patient delay associated with poor TB knowledge has also been described in other studies [5,18,27,31]. In this study, 37.6% of the patients said that they delayed seeking health care because they thought their symptoms would disappear. This finding is consistent with findings from other studies [12,22,31] and shows that some patients wait until symptoms become severe before seeking health care.

The increased delay among patients with an underlying chronic disease can be explained by the failure to recognize TB symptoms. Patients may relate the symptoms to their underlying chronic condition. The insidious character of TB may also contribute to the delay in this group of patients.

HIV infection was associated with shorter patient delay, a finding similar to a study carried out in Thailand [35]. It is possible that patients with HIV infection are more educated about TB, as TB information is actively provided to all patients attending HIV-related services. In fact, in this study the proportion of patients with adequate TB knowledge was higher among HIV-positive patients compared to HIV-negative patients.

### Health system delay

The median health system delay of 62 days was long and similar to that reported in Ghana [28] and Uganda [29]. The duration of health system delay was long even among new smear-positive patients (with a median of 52 days), indicating a failure of the health care system to promptly initiate TB treatment.

The increased health system delay observed among farmers in our study may be explained by the difficulties in visiting the health facilities for follow up visits and to collect the results of diagnostic tests. The association between multiple visits to a health facility and health system delay have been described in several studies [16,26,29,34,36]. Low level of clinical suspicion [29], poor satisfaction with the health care received [26], and delays in obtaining proper laboratory analysis [37] are some of the possible explanations for the delay associated with repeated visits to health facilities. In addition, patients with multiple visits tend to visit different health providers in different health facilities [26,29] making it difficult to reach a prompt TB diagnosis. It is difficult to ascribe cause and effect between delay and multiple visits, as multiple visits necessarily requires the passage of time between the visits. Despite that, multiple visits to a health facility provides a good indication that the healthcare system is failing to diagnosing TB as health workers should be able to suspect TB in any patient with chronic cough and a history of multiple visits to health facilities.

Increased health system delay was also associated with coexistence of an underlying chronic condition. Patients with multiple clinical conditions may present with complex symptoms making it difficult for the health worker to reach a TB diagnosis. This highlights the importance of suspecting TB in any patient with chronic respiratory symptoms, even if the patient has a known underlying condition. It also highlights the need for better TB diagnostic tools.

The present study had some limitations that should be taken in consideration in the interpretation of results. Most importantly, the duration of patient delay may have been underestimated due to poor patient recall of the onset of symptoms, their duration and the date of the first health-seeking encounter. In addition, TB is a chronic disease with an insidious start, making it difficult for patients to remember exactly when the symptoms started.

## Conclusions

This study revealed a long patient and health system delay among patients with pulmonary TB, with a similar contribution of patient delay and health system delay. To reduce patient delay it is crucial to improve TB knowledge, address TB misconceptions, ensure the involvement of traditional healers and pay closer attention to disadvantaged groups such as farmers. Health system delay can be reduced by improving the skills of nurses and medical assistants in TB diagnosis in order to avoid the need for repeated visits to health facilities by patients.

## Competing interests

The authors declare that they have no competing interests.

## Authors’ contributions

AS and JB participated in the design of the study, statistical analysis and preparation of the manuscript. PSG and MS participated in the design of the study and preparation of the manuscript. All authors read and approved the final manuscript.

## Pre-publication history

The pre-publication history for this paper can be accessed here:

http://www.biomedcentral.com/1471-2458/13/559/prepub

## Supplementary Material

Additional file 1 List of questions included in the questionnaire. Click here for file
