# Supplementary material for: Patient and health system delay among patients with pulmonary tuberculosis in Beira city, Mozambique
Source: BMC Public Health. 2013 Jun 7;13:559. doi: 10.1186/1471-2458-13-559 (PMC3680113; doi:10.1186/1471-2458-13-559)
Supplement: Additional file 1 — List of questions included in the questionnaire. [file 1471-2458-13-559-S1.doc]

**List of questions included in the questionnaire**

**Patient identification:**

1. Name of health facility
2. Patient’s name
3. TB Program identification number
4. Patient’s identification number (to be generated in this study)
5. Mobile phone

**Demographic and socio-economic characteristics:**

1. Age
2. Marital status
3. Place of residence
4. Level of education
5. Type of occupation (prior to the disease)
6. Distance (in km) to the nearest health facility
7. Time to reach the nearest health facility from home

**Patient’s TB knowledge and adherence to treatment:**

1. Have you previously heard about TB?
2. Source of information on TB
3. Correctness of knowledge about TB
4. Do you think you will be able to complete the 6 month treatment

**Clinical history:**

1. History of previous TB treatment
2. History of TB household contact
3. History of smoking
4. Number of days that smoked in the past 30 days
5. Number of cigarettes smoked per day in the past 30 days
6. Number of occasions that had an alcoholic beverage in the past 30 days
7. Number of times that had at least 5 “drinks”* in a row
8. Co-existence of other chronic diseases (is patient receiving treatment for HIV/AIDS, Diabetes, COPD, Disability, etc)
9. History of hospital admissions
10. History of imprisonment
11. Height
12. Weight
13. Presence and duration of cough
14. Presence and duration of fever
15. Presence and duration of weight loss
16. Presence and duration of haemoptysis
17. Presence and duration of chest pain
18. Presence and duration of difficulty breathing
19. Presence and duration of night sweats
20. Which symptoms made patient seek healthcare
21. Who patient consulted first
22. Date of first consultation
23. Date first seen by a professional healthcare provider (PHCP)
24. Type of health facility where patient was first seen by a PHCP
25. Reasons for choice of first consultation place
26. Reasons for non-consultation with a public health facility
27. Perceived causes of delay in seeking health care
28. TB stigma (shame, relationships with others, etc)
29. Number of visits before testing was done
30. Number of tests done and results
31. Date of TB diagnosis (determined by sputum result of date of referral to TB clinic)
32. Type of health facility where first diagnosis was made
33. Specialty of the PHCP who made initial diagnosis
34. Laboratory tests and results (sputum smear and HIV)
35. Date of initiation of treatment
36. Date of recording
37. Responsible Officer

* A “drink” means a 12-ounce can (or bottle) of beer, a 4-ounce glass of wine, a 12-ounce bottle (or can) of wine cooler, or a mixed drink or shot of liquor.
